# Prevalence of work-related musculoskeletal disorders among beach workers

**DOI:** 10.3389/fpubh.2025.1701654

**Published:** 2025-12-08

**Authors:** Mariela Sousa dos Santos, Jailma dos Santos Silva, Wiler de Paula Dias, Thayane Silva Nunes, Joelma Marques Rodrigues, Adedayo Michael Awoniyi, Cleber Cremonese

**Affiliations:** 1 Institute of Collective Health, Federal University of Bahia, Salvador, Brazil; 2 Faculty of Medicine of Bahia, Federal University of Bahia, Salvador, Brazil

**Keywords:** epidemiology, cross-sectional studies, workers, beaches, musculoskeletal disorder, sex distribution

## Abstract

**Objective:**

To evaluate the prevalence of repetitive strain injuries (RSIs)/work-related musculoskeletal disorders (WMSDs) and their potential demographic, economic and occupational determinants among urban beach workers in Salvador, Bahia, Brazil, between 2023 and 2024.

**Methods:**

We performed a cross-sectional epidemiological study among 579 urban beach workers in Salvador between November 2023 and March 2024. Following ethical approval and informed consent, structured questionnaires were administered to obtain data on sociodemographic, occupational and work environment characteristics. The outcome, self-reported cases of RSI/WMSDs in the previous 12 months was evaluated using the question “in the last 12 months, have you been diagnosed with RSI/WMSDs?” (yes/no). Prevalence and prevalence ratios were calculated using Poisson regression with robust variance, and analyses were stratified by sex.

**Results:**

The 12-months prevalence of RSI/WMSD was 18.1%. Among participants, 59.4% were male, 25% were aged ≤ 29 years and 11.4% were aged ≥60 years. A total of 92.9% identified as Black or Brown. Informality was reported by 72.3% of workers, 70.2% worked ≥9 h/day and 88% had never received professional training. Women were 1.67 (95% CI: 1.11–2.52) times more likely to report RSI/WMSD compared to men. Working ≥13 h/day increased likelihood of RSI/WMSD by 2.27 (95% CI: 1.12–4.59) times compared with working ≤ 8 h/day. Having experienced at least one work-related accident (WA) in the past year increased the probability of an RSI/WMSD diagnosis by 64% (95% CI: 1.09–2.49).

**Conclusions:**

The overall prevalence of RSI/WMSDs was 18.1%. Female sex, extended working hours and recent WA were associated with the outcome. Although no statistically significant associations were found with broader work conditions, the findings highlighted a precarious occupational context characterized by informality, physical overload, insufficient infrastructure and lack of professional training.

## Introduction

Repetitive Strain Injury (RSIs)/Work-Related Musculoskeletal Disorder (WMSDs) encompass a spectrum of conditions that affect the muscles, nerves, tendons, ligaments, joints, cartilage, intervertebral disks, and other components of the musculoskeletal system (1). Common clinical manifestations include tendonitis, particularly in the shoulders, elbows and wrists, as well as low back pain and diffuse myalgia (2). These conditions are multifactorial in origin, often influenced by individual characteristics such as sex, age and race/skin color and by occupational risk factors, notably ergonomic stressors, repetitive movements, excessive workload and exposure to vibration. Their consequences may range from reduced functional capacity to temporary or permanent work disability, imposing significant health and economic burdens (3–5).

In Brazil, 97,627 cases of RSI/WMSD were reported in the Notifiable Injury Information System (Sistema de Informações de Agravos de Notificação- SINAN) between 2014 and 2024, representing a 64.5% increase over the decade, from 8,341 cases in 2014 to 13,713 in 2024. The highest number of notifications occurred in the Southeast (43,668) and Northeast (25,035) regions. Within the northeastern, Bahia, the state where this study was conducted, accounted for the largest share (*n* = 11,452; 45.7%), followed by Pernambuco (*n* = 4,602; 18.4%) and Ceará (*n* = 3,608; 14.4%) (6). Comparable trends have been documented in other developing countries, such as China (7), and developed countries, including South Korea (8) and Japan (9), underscoring the global relevance of RSI/WMSDs.

RSI/WMSDs may result from inadequately performed work activities, particularly those that exceed the biomechanical limits of the human body and are further influenced by individual predispositions of genetic or hereditary origin (10). Among informal workers, the industrial and commercial sectors generally exhibit high prevalence (11), especially in occupations such as shellfish gatherers (94.7%) (12), bus drivers (65.7%) (13), harvesting machine operators (62.9%) (14) and kitchen workers (51.9%) (15). On the other hand, considerable burden has been observed among formal workers, with Demissie et al. (16) reporting prevalence ranging from 33.8 to 95.3% among bankers and office workers in a systematic review focused mainly on studies from Africa and Asia. Govaerts et al. (17) also reported prevalence between 42 and 60% in a systematic review and meta-analysis of European studies, among industrial workers.

Beyond occupational exposures, sociodemographic characteristics also play a significant role: female gender, Black and Yellow race/color and being within the economically active age group are factors associated with the development of RSI/WMSDs (11). Population-based studies such as this help to elucidate the health conditions of vulnerable groups, such as beach workers, who are often employed under informal and precarious conditions, a common reality in Brazil and other low- and middle-income countries with similar socioeconomic contexts. Estimating RSI/WMSD morbidity in this population provides essential theoretical basis for discussing both individual and economic impacts and for promoting actions aimed at safeguarding workers' quality of life, productivity, and family economic wellbeing.

Despite the substantial body of literature on RSI/WMSD in different occupational groups, important knowledge gaps remain. Methodological challenges often hinder investigations among informal, temporary, and outsourced workers, as well as those employed in remote urban areas. Consequently, limited evidence exists on the burden of RSI/WMSDs among beach workers, a population particularly vulnerable due to precarious employment, lack of formal protection, and adverse working conditions (18). The scarcity of epidemiological data, combined with the apparent neglect of these workers by government institutions, contributes to their invisibility with respect to occupational health. Given this context, this study aimed to evaluate the prevalence of RSI/WMSDs, and their potential demographic, economic and occupational determinants among urban beach workers in Salvador, Bahia, Brazil, between 2023 and 2024.

## Methods

Complete details on the sampling process, collection points (with map), inclusion criteria, and collection procedures can be found in Cremonese et al. (18). The present article describes only the methodological aspects directly relevant to the study objectives.

### Study design, population and study area

This observational, cross-sectional epidemiological survey was conducted among workers engaged in any type of economic activity along the sandy strips of urban beaches in Salvador, Bahia, Brazil. As previously described by Cremonese et al. (18), participants (beach workers) principally engage in activities such as selling beverages, food, clothing, handicrafts, and beauty products, as well as providing services like chair, umbrella and toy rentals, among other petty activities such as surfing, swimming, and diving instructors, waiters, and lifeguards. Five beaches with the highest concentration of workers were selected. For this study, beach was defined as the shoreline area covered or uncovered by water, including the adjacent sand strip, gravel, pebbles, or boulders up to the beginning of natural vegetation begins or, in its absence, another ecosystem (19).

### Sampling process

The target population was defined as 8,355 workers, corresponding to the number of street vendors registered to work during the 2023 Carnival in Salvador, the city's largest annual festival. Based on this information, the minimum sample size was estimated using Epi Info™ software version 7.2.7 from the Centers for Disease Control and Prevention, assuming a 30% prevalence of work-related accidents (WAs), a 95% confidence level, and a 5% margin of error. The calculation indicated a required minimum of 315 workers, with at least 63 from each of the five selected beach areas. Worker selection followed two approaches. For mobile workers carrying out economic activities along the beach, interviewers positioned themselves at central fixed points, and systematically approached every third eligible worker passing by. For workers in fixed locations, interviewers traversed the full length of the beach, identifying workstations, and inviting all workers at every third eligible workstation to participate in the study (18).

### Inclusion and exclusion criteria

Workers were eligible if they were at least 14 years of age, had been engaged in selling products or services on the beaches for a minimum of 3 months, worked at least once per week, and provided informed consent. Individuals were excluded if they had disabilities that prevented communication with researchers, were visibly under the influence of alcohol or psychoactive substances at the time of data collection or withdrew consent or assent during or after participation.

### Data collection procedure

Prior to data collection, procedures were refined through a two-step process: a pre-pilot phase, in which protocols were tested internally among the interviewers, and a pilot phase conducted between August and October 2023 with six urban beach workers in Salvador, who were not included in the final sample.

Interviewers wore personalized project uniforms for easy identification, and conducted interviews in appropriate, accessible locations. Data were collected three times per week (Mondays, Wednesdays, and Fridays) during the morning shift (8:00 a.m. to 12:00 p.m.) between November 6, 2023, and March 8, 2024. A team of eight trained interviewers administered standardized and validated questionnaires using the Research Electronic Data Capture (REDCap) platform, a secure web-based application designed for data collection and management. REDCap allows safe data storage and facilitates export to statistical software such as SPSS, R, Stata, and EpiInfo, among others (20).

The final database was securely stored on a private server managed by the Institute of Collective Health (ISC) of the Federal University of Bahia (UFBA), with access restricted to the research coordinator. All procedures complied with Brazil's General Data Protection Law (Lei Geral de Proteção de Dados- LGPD), ensuring strict confidentiality and security of participants' information (21).

### Independent variables

To characterize the sociodemographic and occupational profile of participants, the following variables were assessed: sex (male/female), age group (≥60, 50–59, 40–49, 30–39, ≤ 29 years); race/ethnicity (Black, Brown, White, Yellow/Indigenous); marital status (married/stable union, single, separated/widowed); educational level (incomplete primary education, complete primary education, incomplete secondary education, complete secondary education, incomplete or complete tertiary education); presence of disabilities (no/yes); having children (no/yes); daily income on weekdays (USD: <14, 14–24, 25–41, and >41), and weekends (USD: <14, 14–24, 25–41, and >41); whether beach work was the participant's sole source of income (no/yes); daily working hours (≤ 8, 9–10, 11–12, ≥13 h); working days per week (≤ 5, 6, 7 days); total months of beach work (≤ 24, 25–96, 97–240, ≥241); current employment condition on the beach (permanent, temporary, emergency due to job loss); currently contribute to the National Institute of Social Security (INSS; no/yes); primary economic activity on the beach (food preparation- stationary, waiter/waitress, sales/service provision- mobile, others); nature of economic activity (stationary -sitting or standing, walking/moving, mixed); carrying weights during work (no/yes); use of personal protective equipment (PPE; no/yes); receipt of training for beach work (no/yes); receipt of assistance from CEREST (Occupational Health Reference Center; no/yes); receipt of any type of government benefits (no/yes); absence of a designated rest space (no/yes); lack of proper storage facilities (no/yes); and occurrence of work-related or commuting accidents in the past 12 months (no/yes).

### Outcome variable

The primary outcome was the presence of a self-reported medical diagnosis of RSI/WMSD [usually grouped together in Brazil notification system (6)] within the past 12 months. This information was obtained through the question: “In the past 12 months, have you been diagnosed with RSI or WMSDs?” Responses were recorded dichotomously (no/yes).

### Data analysis

The sociodemographic and occupational characteristics of participants, general, and stratified by sex, were described using categorized variables, presented as absolute, and relative frequencies (Tables 1, 2). Bivariate analyses were conducted to estimate the prevalence of RSI/WMSDs across categories of independent variables (Tables 3, 4). The chi-square test for heterogeneity was applied to assess statistically significant differences between observed and expected proportions. Variables with *p* ≤ 0.20 in bivariate analysis were considered eligible for inclusion in the multivariable analysis. Crude and adjusted prevalence ratios with 95% confidence intervals were estimated using Poisson regression models with robust variance. In the final model, a significance level of 5% (*p* ≤ 0.05) was adopted to identify associations between sociodemographic and occupational variables and the occurrence of RSI/WMSDs. All analyses were performed using R software, version 4.3.3 (R Project for Statistical Computing). The complete dataset and codes used in the study are publicly available in the Zenodo repository under a Creative Commons license (22) through https://doi.org/10.5281/zenodo.16636781.

**Table 1:** Distribution of sociodemographic and socioeconomic characteristics among urban beach workers in Salvador, BA (2023–2024), by general population and sex (*n* = 579)^*^.

**Variables**	**General sample**		**Sex**		*p*-value^**^
	***n***	**%**	**Female** ***n*** **(%)**	**Male** ***n*** **(%)**	
**Sex**					
Male	343	59.4	–	–	–
Female	234	40.6	–	–	
**Age range**					
≥60	66	11.4	27 (11.5)	39 (11.4)	0.77
59–50	124	21.4	50 (21.4)	74 (21.6)	
49–40	113	19.5	41 (17.5)	72 (21.0)	
39–30	135	23.3	54 (23.1)	81 (23.6)	
≤ 29	141	24.4	62 (26.5)	77 (22.4)	
**Race/color**					
Black	320	55.4	135 (57.7)	184 (53.9)	0.12
Brown	217	37.5	89 (38.0)	127 (37.1)	
White	30	5.2	6 (2.6)	24 (7.0)	
Yellow and indigenous	11	1.9	4 (1.7)	7 (2.0)	
**Marital status**					
Married or in a stable union	167	28.8	54 (23.1)	112 (32.7)	0.02
Single	371	64.1	158 (67.5)	212 (61.8)	
Separated or widowed	41	7.1	22 (9.4)	19 (5.5)	
**Educational level**					
Tertiary education (incomplete or completed)	23	4.1	6 (2.6)	17 (5.2)	0.05
Secondary education (completed)	209	37.1	41 (17.8)	109 (32.9)	
Secondary education (incomplete)	94	16.7	99 (43.0)	52 (15.7)	
Primary education (completed)	70	12.4	26 (11.4)	44 (13.3)	
Primary education (incomplete)	167	29.7	58 (25.2)	109 (32.9)	
**Person with disability**					
No	537	94.5	220 (95.2)	315 (94.0)	0.66
Yes	31	5.5	11 (4.8)	20 (6.0)	
**Has children**					
No	175	30.3	59 (25.2)	114 (33.3)	0.04
Yes	403	69.7	175 (74.8)	228 (66.7)	
**Daily income/weekdays in dollars** ^***^					
>41	136	24.9	53 (24.3)	83 (25.5)	0.53
41–25	136	24.9	48 (22.0)	87 (26.7)	
24–14	136	24.9	59 (27.1)	76 (23.3)	
<14	138	25.3	58 (26.6)	80 (24.5)	
**Daily/weekend income in dollars** ^***^					
>41	136	24.9	56 (25.5)	80 (24.6)	0.03
41–25	132	24.1	40 (18.2)	91 (28.1)	
24–14	113	20.7	74 (24.5)	58 (17.8)	
<14	166	30.3	70 (31.8)	96 (29.5)	
**Is beach work your sole source of income?**					
No	151	26.2	60 (25.8)	91 (26.6)	0.89
Yes	426	73.8	173 (74.2)	251 (73.4)	

^*^The sum of some categories is <579 due to lack of responses.

^**^Chi-square test for heterogeneity–statistical significance defined as *p* < 0.05.

^***^Division into quartiles. NB: average exchange rate at time of data collection–USD 1 = R$ 4.90.

**Table 2:** Distribution of occupational and work environment characteristics among urban beach workers in Salvador, BA (2023–2024), by general population and sex (*n* = 579)^*^.

**Variables**	**General sample**		**Sex**		*p*-value^**^
	***n***	**%**	**Female** ***n*** **(%)**	**Male** ***n*** **(%)**	
**No. of hours spent working on the beach per day**					
≤ 8	171	29.8	68 (29.1)	102 (30.3)	0.98
9–10	177	30.9	72 (30.8)	104 (30.9)	
11–12	174	30.4	73 (31.2)	101 (30.0)	
≥13	51	8.9	21 (8.9)	30 (8.8)	
**No. of days spent working on the beach per week**					
≤ 5	186	32.4	76 (32.6)	109 (32.2)	0.98
6	186	32.4	76 (32.6)	110 (32.4)	
7	202	35.2	81 (34.8)	120 (35.4)	
**Total time spent working on the beach (in months)**					
≤ 24	164	28.4	81 (34.6)	81 (23.6)	0.01
25–96	133	23.0	44 (18.9)	89 (25.9)	
97–240	161	27.9	65 (28.0)	96 (28.1)	
≥241	120	20.7	43 (18.5)	77 (22.4)	
**Current work condition on the beach**					
Permanent	189	32.8	91 (39.1)	97 (28.5)	0.02
Temporary (supplementary job)	117	20.3	46 (19.7)	70 (20.5)	
Emergency (due to job loss)	270	46.9	96 (41.2)	174 (51.0)	
**Currently contributes to INSS**					
Yes (CLT/MEI/PJ)	160	27.7	64 (27.4)	95 (27.9)	0.96
No	417	72.3	170 (72.6)	246 (72.1)	
**Primary economic activity on the beach**					
Food preparation and/or sale (stationary)	131	22.7	62 (26.6)	68 (19.9)	0.02
Waiter/waitress	144	25.0	54 (23.2)	89 (26.1)	
Sales and service provision (moving)	251	43.6	105 (45.1)	146 (42.8)	
Others	50	8.7	12 (5.1)	38 (11.2)	
**Characteristics of economic activity**					
Stationary (sitting or standing)	107	18.6	64 (27.2)	43 (12.6)	<0.001
Walking/moving	267	46.4	86 (36.8)	180 (52.9)	
Mixed (stationary and moving)	202	35.0	84 (36.0)	117 (34.5)	
**Works by carrying weight**					
No	155	27.0	83 (35.5)	72 (21.2)	<0.001
Yes	420	73.0	151 (64.5)	267 (78.8)	
**Works using PPE**					
Yes	201	35.1	79 (34.1)	121 (35.8)	0.73
No	371	64.9	153 (65.9)	217 (64.2)	
**Received training to work on the beach**					
Yes	79	13.9	197 (85.3)	292 (86.6)	0.99
No	491	86.1	37 (14.7)	45 (13.4)	
**Received any assistance from Cerest?**					
Yes	36	6.3	11 (4.7)	25 (7.4)	0.25
No	538	93.7	223 (95.3)	313 (92.6)	
**Received any type of government benefits (municipal, state or federal)**					
Yes	268	46.9	136 (58.4)	130 (38.7)	<0.001
No	303	53.1	97 (41.6)	206 (61.3)	
**Lack of a designated rest space**					
No	200	35.0	85 (37.0)	114 (33.6)	0.46
Yes	371	65.0	145 (63.0)	225 (66.4)	
**Lack of proper storage facilities for equipment**					
No	125	21.9	42 (18.3)	82 (24.1)	0.11
Yes	447	78.1	188 (81.7)	258 (75.9)	
**Work accident/route** ≤ **12 months**					
No	344	59.7	136 (58.1)	207 (60.9)	0.56
Yes	232	40.3	98 (41.9)	133 (39.1)	
**Diagnosis of RSI/WRMD** ^***^					
No	471	81.9	175 (75.1)	294 (86.5)	<0.001
Yes	104	18.1	58 (24.9)	46 (13.5)	

^*^The sum of some categories is <579 due to lack of responses.

^**^Chi-Square test for heterogeneity–statistical significance defined as *p* < 0.05.

^***^Repetitive strain injury/workplace-related musculoskeletal disorders (RSI/WRMD).

INSS, National Institute of Social Security; PPE, personal protective equipment; Cerest, Occupational Health Reference Center.

**Table 3:** Prevalence (P) and crude prevalence ratio (CPR) of RSI/WRMD^*^ diagnoses in the past 12 months among urban beach workers in Salvador, BA (2023–2024), general population and sex (*n* = 579)^**^.

**Variables**	**General sample**		**Sex**			
			**Female**		**Male**	
	***P***	**CPR (CI 95%)**	***P***	**CPR (CI 95%)**	***P***	**CPR (CI 95%)**
**Sex**						
Male	13.5	1.00	–	–	–	–
Female	24.8	1.84 (1.25–2.72)	–	–	–	–
**Age range**						
≥60	24.3	1.00	33.3	1.00	17.9	1.00
59–50	16.3	0.67 (0.35–1.31)	20.0	0.60 (0.24–1.51)	13.7	0.76 (0.29–2.10)
49–40	16.1	0.66 (0.34–1.31)	24.3	0.73 (0.29–1.84)	11.3	0.63 (0.23–1.79)
39–30	14.8	0.61 (0.32–1.20)	27.8	0.83 (0.37–1.98)	6.2	0.34 (0.10–1.08)
≤ 29	21.5	0.89 (0.49–1.67)	22.9	0.69 (0.30–1.65)	21.0	1.17 (0.50–3.05)
**Race/color**						
Black	19.5	1.00	27.6	1.00	13.7	1.00
Brown	16.7	0.85 (0.56–1.28)	21.3	0.77 (0.44–1.33)	13.5	0.98 (0.52–1.81)
White	13.3	0.68 (0.21–1.65)	16.7	0.60 (0.03–2.78)	12.5	0.91 (0.22–2.59)
Yellow and indigenous	18.2	0.93 (0.15–2.97)	25.0	0.91 (0.05–4.17)	14.3	1.04 (0.06–4.90)
**Marital status**						
Married or in a stable union	19.2	1.00	25.9	1.00	25.9	1.00
Single	17.2	0.90 (0.59–1.39)	24.8	0.96 (0.53–1.83)	24.8	0.71 (0.39–1.33)
Separated or widowed	21.9	1.15 (0.51–2.30)	22.7	0.88 (0.28–2.29)	22.7	1.31 (0.38–3.51)
**Educational level**						
Tertiary education (incomplete or completed)	18.2	1.00	16.7	1.00	18.7	1.00
Secondary education (completed)	23.4	1.29 (0.53–4.27)	21.9	1.32 (0.25–24.29)	15.6	0.83 (0.28–3.56)
Secondary education (incomplete)	19.3	1.06 (0.40–3.69)	21.9	1.32 (0.25–24.29)	17.6	0.94 (0.28–4.24)
Primary education (completed)	21.4	1.18 (0.43–4.13)	30.8	1.85 (0.34–34.25)	15.9	0.85 (0.24–3.94)
Primary education (incomplete)	9.1	0.50 (0.18–1.75)	10.5	0.63 (0.11–11.93)	8.3	0.44 (0.13–2.00)
**Person with disability**						
No	18.2	1.00	24.6	1.00	13.8	1.00
Yes	22.6	1.24 (0.52–2.48)	36.3	1.47 (0.45–3.60)	15.0	1.09 (0.26–2.98)
**Has children**						
No	17.9	1.00	25.4	1.00	14.3	1.00
Yes	18.2	1.02 (0.67–1.57)	24.7	0.97 (0.55–1.81)	13.2	0.93 (0.51–1.74)
**Daily income/weekdays in dollars** ^***^						
>41	17.0	1.00	20.7	1.00	14.6	1.00
41–25	16.9	0.99 (0.55–1.48)	29.2	1.41 (0.64–3.17)	10.3	0.71 (0.29–1.67)
24–14	21.5	1.26 (0.73–2.20)	27.6	1.33 (0.62–2.95)	17.1	1.17 (0.53–2.60)
<14	15.2	0.89 (0.49–1.62)	22.4	1.08 (0.48–2.46)	10.0	0.68 (0.27–1.65)
**Daily/weekend income in dollars** ^***^						
>41	20.7	1.00	25.0	1.00	17.7	1.00
41–25	12.1	0.58 (0.31 1.07)	22.5	0.90 (0.37–2.05)	7.7	0.43 (0.16–1.04)
24–14	19.6	0.95 (0.54–1.95)	22.6	0.91 (0.41–1.96)	17.2	0.97 (0.42–2.17)
<14	18.7	0.90 (0.54–1.51)	27.1	1.09 (0.55–2.21)	12.5	0.71 (0.32–1.53)
**Is beach work your sole source of income?**						
No	20.8	1.00	26.7	1.00	16.8	1.00
Yes	17.2	0.83 (0.55–1.27)	24.1	0.92 (0.53–1.68)	12.3	0.73 (0.40–1.40)

^*^Repetitive strain injury/work-related musculoskeletal disorder (RSI/WRMD).

^**^The sum of some categories is <579 due to lack of responses.

^***^Division into quartiles. NB: average exchange rate at time of data collection–USD 1 = R$ 4.90.

CI, confidence interval 95%.

Value 1.00 = reference category.

**Table 4:** Prevalence (P) and crude prevalence ratio (CPR) of RSI/WMSD^*^ diagnoses in the past 12 months among urban beach workers in Salvador, BA (2023–2024), by general population, sex and according to occupational and work environment characteristics (*n* = 579)^**^.

**Variables**	**General sample**		**Sex**			
			**Female**		**Male**	
	***P***	**CPR (IC 95%)**	***P***	**CPR (CI 95%)**	***P***	**CPR (CI 95%)**
**No. of hours spent working on the beach per day**						
≤ 8	11.2	1.00	13.2	1.00	9.9	1.00
9–10	17.5	1.57 (0.89–2.82)	23.6	1.78 (0.81–4.19)	13.4	1.36 (0.61–3.15)
11–12	21.3	1.90 (1.11–3.38)	32.9	2.48 (1.20–5.65)	12.9	1.30 (0.57–3.05)
≥13	34.0	3.04 (1.57–5.86)	40.0	3.02 (1.13–7.90)	30.0	3.03 (1.20–7.52)
**No. of days spent working on the beach per week**						
≤ 5	17.4	1.00	21.0	1.00	15.6	1.00
6	17.3	0.97 (0.60–159)	24.0	1.14 (0.58–2.26)	12.7	0.82 (0.40–1.65)
7	19.4	1.09 (0.69–1.75)	29.6	1.41 (0.75–2.70)	12.6	0.81 (0.40–1.62)
**Total time spent working on the beach (in months)**						
≤ 24	17.1	1.00	21.0	1.00	13.6	1.00
25–96	16.9	0.99 (0.56–1.73)	20.4	0.97 (0.42–2.14)	15.1	1.11 (0.50–2.53)
97–240	18.1	1.06 (0.63–1.79)	23.4	1.12 (0.55–2.24)	14.6	1.07 (0.49–2.42)
≥241	20.8	1.22 (0.71–2.09)	39.5	1.88 (0.96–3.71)	10.4	0.77 (0.30–1.89)
**Current work condition on the beach**						
Permanent	16.2	1.00	31.1	1.00	11.3	1.00
Temporary (supplementary job)	20.7	0.78 (0.44–1.34)	17.4	0.56 (0.24–1.17)	15.7	1.39 (0.59–3.24)
Emergency (due to job loss)	16.8	0.81 (0.53–1.25)	22.9	0.74 (0.42–1.28)	13.4	1.19 (0.59–2.53)
**Currently contributes to INSS**						
Yes (CLT/MEI/PJ)	18.1	1.00	26.5	1.00	12.6	1.00
No	18.1	1.00 (0.66–1.55)	24.2	0.91 (0.53–1.65)	13.9	1.10 (0.59–2.21)
**Primary economic activity on the beach**						
Food preparation and/or sale (stationary)	19.8	1.00	27.4	1.00	13.2	1.00
Waiter/Waitress	21.8	1.10 (0.65–1.87)	28.3	1.03 (0.51–2.07)	18.2	1.37 (0.62–3.25)
Sales and service provision (moving)	15.5	0.78 (0.48–1.30)	21.9	0.80 (0.43–1.52)	10.9	0.83 (0.37–1.96)
Others	16.0	0.81 (0.34–1.70)	25.0	0.91 (0.21–2.71)	13.1	0.99 (0.31–2.88)
**Characteristics of economic activity**						
Stationary (sitting or standing)	18.7	1.00	23.4	1.00	11.6	1.00
Walking/moving	18.3	0.98 (0.59–1.69)	29.1	1.24 (0.66–2.41)	13.3	1.15 (0.48–3.41)
Mixed (stationary and moving)	17.4	0.93 (0.54–1.64)	21.7	0.93 (0.47–1.86)	14.5	1.25 (0.49–3.80)
**Works by carrying weight**						
No	12.2	1.00	13.2	1.00	11.1	1.00
Yes	20.1	1.64 (1.02–2.78)	31.3	2.36 (1.27–4.80)	13.9	1.25 (0.61–2.90)
**Works using PPE**						
Yes	18.6	1.00	23.5	1.00	15.2	1.00
No	17.5	0.94 (0.62–1.40)	28.2	1.20 (0.70–2.02)	10.7	0.71 (0.36–1.31)
**Received training to work on the beach**						
Yes	41.6	1.00	42.8	1.00	40.0	1.00
No	16.4	1.07 (0.60–2.12)	22.2	1.10 (0.51–2.88)	12.5	1.04 (0.45–3.02)
**Received any assistance from Cerest?**						
Yes	13.9	1.00	18.2	1.00	0.12.0	1.00
No	18.4	1.33 (0.60–3.76)	25.2	1.39 (0.43–8.47)	13.7	1.14 (0.42–4.72)
**Received any type of government benefits (municipal, state or federal)**						
Yes	16.8	1.00	23.7	1.00	10.0	1.00
No	19.1	1.14 (0.77–1.68)	25.8	1.09 (0.64–1.83)	16.0	1.60 (0.86–3.16)
**Lack of a designated rest space**						
No	14.1	1.00	16.7	1.00	12.3	1.00
Yes	19.9	1.42 (0.93–2.23)	29.0	1.74 (0.97–3.30)	14.2	1.16 (0.63–2.24)
**Lack of proper storage facilities for equipment**						
No	9.6	1.00	11.9	1.00	8.5	1.00
Yes	20.2	2.10 (1.20–4.04)	27.3	2.29 (1.01–6.59)	15.1	1.77 (0.84–4.33)
**Work accident/route** ≤ **12 months**						
No	13.5	1.00	17.8	1.00	10.6	1.00
Yes	25.0	1.86 (1.27–2.76)	34.7	1.95 (1.16–3.33)	18.0	1.70 (0.95–3.05)

^*^Repetitive strain injury/work-related musculoskeletal disorder (RSI/WRMD).

^**^The sum of some categories is <579 due to lack of responses.

INSS, National Institute of Social Security; PPE, personal protective equipment; Cerest, Occupational Health Reference Center; CI, confidence interval 95%.

Value 1.00 = reference category.

### Ethical procedures

The study was approved by the Brazilian Research Ethics Committee, in accordance with Resolution No. 466/2012, and Operational Standard No. 001/2013 of the National Health Council, under CAAE: 68859623.0.0000.5030, and opinion No. 6,501,806. All participants provided informed consent, agreeing to participate in the study and permitting the anonymous publication of the results, after reading and signing the Free and Informed Consent Form (FICF). For participants under 18 years of age, both the FICF and authorization form signed by a legal guardian were required.

## Results

### General description of participants' sociodemographic and occupational characteristics

Between November 2023 and March 2024, a total of 579 interviews were conducted with workers distributed across five stretches of urban beaches in Salvador, Bahia. Regarding the sociodemographic profile, most participants were male (59.4%), and aged ≤ 49 years (67.2%). The majority self-identified as Black or Brown (92.9%), and reported being single (64.1%). More than half (58.8%) had not completed high school, and a large proportion reported having children (69.7%). Most workers (50.2%) earned less than USD 24.00 per day during weekdays, and 51% reported this level of income on weekends. For 73.8% beach-related work was the sole source of household subsistence (Table 1).

Regarding occupational characteristics 70.2% reported working more than 9 h per day, while 67.6% worked six to seven days per week. Most 71.6% had been engaged in beach-related activities for over 2 years. Informality predominated, as 72.3% reported not contributing to the INSS system. A large proportion (81.4%) reported performing their activities in constant movement, and 73% carried weight throughout the workday. The majority (64.9%) did not use PPE, and 78.1% lacked proper storage facility to store their work materials. In the past 12 months, 40.3% reported experiencing work-related or commuting accidents, with similar proportions among women (41.9%), and men (39.1%). Concerning self-reported diagnosis of RSI/WMSDs, 18.1% workers indicated having received such diagnosis, higher among women (24.9%) compared to men (13.5%; Table 2).

### Bivariable prevalence of RSI/WMSDs among participants

When stratified by sex, higher prevalence of RSI/WMSDs among women was observed in those aged ≥60 years (33.3%), identifying as Black (27.6%), married or in a stable union (25.9%), with educational level up to secondary school (21.9%), reporting disability (36.3%), with children (24.7%), and lower daily income of USD 14.00–24.00 on weekdays (27.6%), and up to USD <14.00 on weekends (27.1%). Prevalence was also elevated among women for whom beach work was not the sole source of income (26.7%). Among men, prevalence was higher in younger workers (21.0%), those identifying as Yellow or Indigenous (14.3%), married or in a stable union (25.9%), and with higher educational levels (18.7%). Greater prevalence was also reported among men with disabilities (15.0%), without children (14.3%), with daily income of USD 14.00–24.00 on weekdays (17.1%), and above of USD ≥41 on weekends (17.7%), as well as those engaged in addition occupations besides beach work (16.8%; Table 3).

With respect to work characteristics, 21.3% of workers reporting RSI/WMSDs in the past year worked 11–12 h per day, corresponding to a crude prevalence ratio (CPR) of 1.90 (95% CI: 1.11–3.38) in the general sample. Stratification by sex revealed that women accounted for 32.9% of cases (CPR: 2.48; 95% CI: 1.20–5.65), whereas men accounted for 12.9% (CPR: 1.30; 95% CI: 0.57–3.05). Although duration of beach work was not statistically significance, prevalence was higher (20.8%) among workers with more than 241 months of activity (CPR: 1.22; 95% CI: 0.71–2.09). In this category, prevalence reached 39.5% among women (CPR: 1.88; 95% CI: 0.96–3.71), and 10.4% among men (CPR: 0.77; 95% CI: 0.30–1.89). Carrying weight was associated with higher prevalence among women (31.3%; CPR: 2.36; 95% CI: 1.27–4.80). Lack of proper storage facilities for equipment was associated with RSI/WMSD among women (CPR: 2.29; 95% CI: 1.01–6.59). Work-related or commuting accidents were associated with 25.0% prevalence overall (CPR: 1.86; 95% CI: 1.27–2.76). Sex-stratified analysis showed higher prevalence among women (34.7%; CPR:1.95; 95% CI: 1.16–3.33) than among men (18%; CPR: 1.70; 95% CI: 0.95–3.05; Table 4).

### Adjusted multivariable prevalence of RSI/WMSDs among participants

The adjusted prevalence ratio (aPR) for the diagnosis of RSI/WMSD in the past 12 months, for the general sample and stratified by sex, is presented in Table 5. Overall, female workers exhibited a significantly higher prevalence of RSI/WMSD compared to male workers (aPR: 1.67; 95% CI: 1.11–2.52). Longer working hours per day were associated with an increased probability of RSI/WMSDs in the overall sample (aPR: 2.27; 95% CI: 1.12–4.59). Additionally, a history of work-related accidents was significantly associated with higher prevalence in general sample (aPR: 1.67; 95% CI: 1.09–2.49).

**Table 5:** Adjusted prevalence ratio (aPR) of RSI/WMSD^*^ diagnosis in the past 12 months among urban beach workers in Salvador, BA (2023–2024), by general population and sex (*n* = 579)^**^.

**Variables**	**aPR (CI 95%)**		
	**General sample**	**Sex**	
		**Female**	**Male**
**Sex**			
Male	1.00	–	–
Female	1.67 (1.11–2.52)	–	–
**Age range**			
≥60	–	–	1.00
50–59	–	–	0.97 (0.32–3.25)
40–49	–	–	0.81 (0.26–2.80)
30–39	–	–	0.38 (0.10–1.42)
≤ 29	–	–	1.33 (0.50–4.17)
**Educational level**			
Tertiary education (incomplete or completed)	1.00	1.00	–
Secondary education (completed)	0.85 (0.34–2.85)	1.32 (0.25–24.45)	–
Secondary education (incomplete)	0.82 (0.30–2.89)	0.94 (0.16–17.93)	–
Primary education (completed)	0.98 (0.35–3.46)	2.00 (0.34–38.10)	–
Primary education (incomplete)	0.39 (0.14–1.39)	0.44 (0.07–8.72)	–
**Daily income/weekdays in dollars** ^***^			
>41	–	–	1.00
41–25	–	–	0.42 (0.16–1.03)
24–14	–	–	0.89 (0.37–2.08)
<14	–	–	0.65 (0.29–1.44)
**No. of hours spent working on the beach per day**			
≤ 8	1.00	1.00	1.00
9–10	1.44 (0.80–2.66)	1.75 (0.75–4.39)	1.52 (0.65–3.73)
11–12	1.66 (0.94–3.06)	2.08 (0.93–5.17)	1.17 (0.49–2.89)
≥13	2.27 (1.12–4.59)	2.06 (0.68–6.17)	2.72 (0.99–7.33)
**Total time spent working on the beach (in months)**			
≤ 24	–	1.00	–
25–96	–	0.82 (0.34–1.86)	–
97–240	–	0.93 (0.42–2.02)	–
≥241	–	1.57 (0.66–3.72)	–
**Current work condition on the beach**			
Permanent	–	1.00	–
Temporary (supplementary job)	–	1.04 (0.53–2.06)	–
Emergency (due to job loss)	–	0.57 (0.21–1.35)	–
**Works by carrying weight**			
No	1.00	1.00	–
Yes	1.55 (0.93–2.73)	1.67 (0.86–3.52)	–
**Received training to work on the beach**			
Yes	–	–	–
No	–	–	–
**Received any type of government benefits (municipal, state or federal)**			
Yes	–	–	1.00
No	–	–	1.37 (0.72–2.75)
**Lack of a designated rest space**			
No	1.00	1.00	–
Yes	1.13 (0.72–1.82)	1.20 (0.63–2.43)	–
**Lack of proper storage facilities for equipment**			
No	1.00	1.00	1.00
Yes	1.68 (0.93–3.31)	1.56 (0.63–4.72)	1.47 (0.68–3.63)
**Work accident/route** ≤ **12 months**			
No	1.00	1.00	1.00
Yes	1.64 (1.09–2.49)	1.66 (0.93–3.04)	1.61 (0.86–3.03)

^*^Repetitive strain injury/work-related musculoskeletal disorder (RSI/WRMD).

^**^The sum of some categories is <579 due to lack of responses.

^***^Division into quartiles. NB: average exchange rate at time of data collection–USD 1 = R$ 4.90.

CI, confidence interval 95%.

Value 1.00 = reference category.

General model–controlling for sex, educational level, number of hours spent working on the beach per day, works by carrying weight, lack of a designated rest space, lack of proper storage facilities for equipment, and work accident/route ≤ 12 months; female model–controlling for educational level, number of hours spent working on the beach per day, total time spent working on the beach (in months), current work condition on the beach, works by carrying weight, lack of a designated rest space, lack of proper storage facilities for equipment, and work accident/route ≤ 12 months; and male model - controlling for age range, daily income/weekdays in dollars^***^, number of hours spent working on the beach per day, received any type of government benefits (municipal, state or federal), lack of proper storage facilities for equipment, and work accident/route ≤ 12 months.

## Discussion

This study assessed the prevalence of RSI/WMSDs and their potential demographic, economic and occupational determinants among urban beach workers in Salvador, Bahia, Brazil, between 2023 and 2024. Here we reported an overall prevalence of 18.1%, with higher probabilities of occurrence among women, workers reporting ≥13 working hours per day and those with a recent history of work-related accidents. The paucity of research specifically addressing RSI/WMSDs among beach workers underscores the importance of comparing these findings with other occupational groups characterized by high degree of informality, precarious working conditions, and limited or absent labor rights. Notably, higher prevalence has been documented in both national and international studies investigating RSI/WMSDs among informal workers, particularly 94.7% among shellfish gatherers (12), 65.7% among bus drivers (13), 62.9% among harvesting machine operators (14), and 51.9% among kitchen workers (15). The comparatively lower prevalence observed here may, in part, be attributable to methodological criteria: RSI/WMSDs were only considered when participants reported a diagnosis confirmed by a health professional, and only cases occurring within the preceding 12 months were included.

Among work-related diseases and injuries, RSI/WMSDs constitute the third principal cause of compulsory notifications, surpassed only by WAs and exposure to biological material (6). According to data from the national health information system, 26,337 cases of RSI/WMSDs were reported between 2023 and 2024, of which 12,242 (46.5%) resulted in temporary removed from work activities. The diagnosis of RSI/WMSDs, as well as the resulting need for temporary or permanent work leave, generates significant emotional, financial, and physical burdens for workers and their families, with repercussions that extend to employers and society at large (4).

Underreporting of RSI/WMSDs remains a persistent challenge for occupational health surveillance. Despite advances in digital health and the expansion of health information systems, such as SINAN, and others specifically targeting worker health, limitations remain in establishing and characterizing the causal link between work activity and RSI/WMSDs (23). In labor markets characterized by high informality and restricted access to both primary health care and specialized occupational health services, as is the case in Brazil, official records are likely to capture only a fraction of the true burden (24, 25).

To explore possible factors associated with the prevalence of RSI/WMSDs, multivariable analysis revealed that individuals working ≥13 h per day were 2.27 times more likely to develop the outcome compared with those working ≤ 8 h per day. The association between prolonged working hours and the development of RSI/WMSDs has been consistently reported in both national and international studies (1, 26, 27). For example, a cross-sectional epidemiological study of 209 fisherwomen/shellfish gatherers in Bahia, whose working conditions closely resemble those of beach workers, found a significantly higher prevalence of RSI/WMSDs among those working ≥8 h per day (12). Similarly, a multivariable analysis of 936 call centers workers in the United Kingdom demonstrated a positive association between workload and RSI/WMSDs (27). These findings align with the argument by Silva andAraújo (5) that the frequency and duration of work activities are key determinants of RSI/WMSDs, as excessive workloads both restrict certain movements and intensify repetitive tasks, thereby contributing to musculoskeletal strain and injury.

With respect to the association between the history of WAs and the diagnosis of RSI/WMSDs, workers who reported at least one type of WA in the past 12 months had 1.86-fold greater probability of the outcome. Although direct evidence linking RSI/WMSDs to an increased likelihood of WAs is limited, a cross-sectional study of 1,304 informal commercial workers in Bahia (2013) identified a significant association between WAs and unsatisfactory ergonomic conditions, including inadequate postures, manual load handling, force exertion, repetitive movements, frequent walking, and standing work (28). Such conditions impose high physical demands, reducing work capacity, and potentially heightened the risk of accidents. Several hypotheses may underlie this association. Workers already diagnosed with RSI/WMSDs who continue working under poor ergonomic conditions may be more susceptible WAs. Conversely, workers with a history of WAs may develop temporary or permanent sequelae that alter their work routines, leading to limb overload, and subsequent development of RSI/WMSDs. It is important to note that, in this study, both conditions were evaluated within a restricted 12-month period. Given the cross-sectional study, casual relationship between these variables cannot be established.

Stratification by sex provided a clearer understanding of the influence of this determinant on the outcome investigated. Overall, women were 67% more likely than men to be diagnosed with RSI/WMSDs. Potential explanatory factors were identified that help explain the higher incidence of RSI/WMSDs among women. From a structural perspective, as noted in previous studies (25, 29), women in informal work are frequently engaged in tasks that demand repetitive movements and prolonged static postures. Here, this pattern was evident in activities such as preparing *acarajé* dough (bean cake fried in hot palm oil), which requires continuous manual effort and repetitive molding. Moreover, economic inequality exacerbates this vulnerability according to the Annual Social Information Report (30), women earn, on average, only 79.3% of the salary received by men in equivalent positions in Brazilian companies. A similar disparity was observed here, where women reported daily average earning of USD 14.00–24.00 compared with USD 25.00–41.00 for men. Such inequalities intensify women's exposure to working conditions that increase the risk of illness.

Biological factors could help explain the higher prevalence of RSI/WMSDs among female workers, as evidenced in this and other national and international studies (1, 31, 32). Beyond direct causal link, multifactorial risk factors may also be involved (33). On average, women have between 30 and 40% less muscle mass than men, 43%−63% lesser strength in the upper-limb, and a predominance of type I muscle fibers, characteristics that limit tolerance to repetitive physical exertion (1, 34). When combined with precarious and informal work conditions, marked by long hours, inadequate or lack of rest breaks, and physical overload, the likelihood of illness increases. In such contexts, women are often engaged in repetitive, physically demanding tasks with little structural support or social support, a scenario that substantially contributes to the development of musculoskeletal disorders, such as RSI/WMSD (25, 29).

In addition the “triple work shift” -which encompasses paid job, domestic chores, and childcare, represents a significant determinant of the physical and emotional overload experienced by women (35). In a study of 158 workers from a furniture factory in São Paulo, women were disproportionately affected, reporting pain attributed not only to the triple work shift but also to their responsibility for more detailed tasks. It is important to note that pain is an early manifestation of RSI/WRMDs, particularly when associated with pathophysiological and psychosocial factors (33). Thus, beyond biological limitations and job insecurity, women experience additional suffering arising from the difficulty in reconciling professional demands with socially prescribed roles, such as childcare and domestic responsibilities (35).

Regarding the method used to measure the outcome of interest “a recent history of RSI/WMSD diagnosis within the past 12 months,” the strategy of directly asking workers about the existence of a medical diagnosis, without requiring the presentation of medical reports, may be considered limited. Nonetheless, this approach is widely used in occupational epidemiology (36–38). It is important to note that RSI/WMSDs are typically diagnosed on the basis of clinical criteria, complemented by additional examinations and by establishing a causal link through the temporal relationship with working conditions. In this study population, composed predominantly of informal workers, access to pre-employment or periodic medical evaluations, common in formal employment settings, was not available. Therefore, despite its limitations, the method adopted represents a standard and accepted practice in epidemiological studies involving workers in informal contexts (39, 40).

It is important to highlight the Regulatory Standard (NR-17), Ergonomics, which establishes guidelines and requirements for adapting working conditions to workers' psychophysiological characteristics, with the aim of promoting comfort, safety, health, and efficiency at work (41). The application of NRs is mandatory for employers operating under formal employment contracts. However, informal workers, unregistered self-employed individuals, and cooperative workers outside the formal labor regime are not legally covered by these regulations. Despite this legal limitation, the ergonomic and occupational health principles contained in NR 17 and other NRs should be extended to all categories of workers. Public managers, through educational initiatives and ergonomic assessments, can develop programs and clinical monitoring tailored to vulnerable sectors, thereby applying the principle of equity.

In the present study, the prevalence of RSI/WMSDs was the same among informal and formal workers (18.1%). According to recent IBGE data, 51% of workers in the state of Bahia are informal, compared to 42% in the state capital, Salvador (42). Although high, these proportions are lower than the 72% informality observed among beach workers in this study. The literature consistently associates informality with greater exposure to ergonomic risks and consequently, with a higher likelihood of developing RSI/WMSDs (32, 43, 44). The absence of this expected trend in our findings warrants further reflection. One possible explanation is that employment relationships, which theoretically reduce the risk of RSI/WMSDs by guarantying safer working conditions, do not always achieve this purpose when safe practices and appropriate conditions are not effectively implemented. In the context of beach-related economic activities, both formal and informal workers are often exposed to equally precarious working conditions. For instance, formal workers registered as individual microentrepreneurs may still work long hours, perform physically demanding tasks, operate in settings with poor or absent ergonomic adaptations, take irregular or no breaks, and lack adequate PPE. These findings suggest that employment status alone is insufficient to protect workers from occupational hazards. In the absence of effective public policies that promote ergonomic infrastructure, systematic breaks, workplace exercise programs and occupational health education initiatives, beach workers, regardless of whether they are formally or informally employed, remain equally vulnerable to RSI/WMSDs.

The concept of precariousness, understood as the progressive deterioration of working conditions, social protection and workers' rights, is clearly reflected in the occupational profile of the workers analyzed in this study (43–45). The weakening of collective organization, insecurity in daily activities and the erosion of occupational identity were evident in our findings and consistent with previous publication (18). These issues are particularly pronounced given the high proportion of informal workers, who face unstable pay and lack fundamental legal guarantees and labor rights, such as paid vacation, a 13th-month salary, sick leave, and social security coverage. Additionally, the beach work environments observed here lacked even the most basic infrastructure, including access to drinking water, restrooms, and designated rest space (46), thereby exposing workers to daily health risks. These conditions are further aggravated by constant exposure to extreme heat, which is intensifying as a result of climate change, which is associated with both acute and chronic health outcomes. Taken together, these adverse conditions identify beach workers as a particularly vulnerable occupational group, requiring urgent interventions. Nevertheless, to the best of our knowledge, the absence of public policies directed toward this workforce, both locally, and nationally, has contributed directly to the worsening of their physical and mental health.

While this study offers insights into the prevalence of RSI/WMSDs among beach workers in Salvador, it is not without limitations. Most notably, as a cross-sectional study, it does not allow for follow-up of participants over time or the assessment of exposures and outcome at multiple time points. To reduce the risk of reverse causality, strategies such as the inclusion of time-specific questions were adopted. For example, participants were asked whether they had been diagnosed with RSI/WMSD in the past 12 months. However, supporting documents, such as medical reports, were not required. Although interviewers emphasized that only professional diagnosed cases from the previous year should be reported, the possibility of overestimating prevalence cannot be ruled out. Regarding the limitation related to the investigated outcome, it is important to note that RSI/MSD was assessed as a single entity, without information on specific anatomical sites (e.g., upper limbs, back, or lower limbs), which somewhat constrains a more detailed interpretation of the results. Another important consideration is the potential for healthy worker bias. Despite the observed high prevalence of RSI/WMSD, it is possible that only workers who were physically and mentally fit to perform economic activities on the beaches at the time of data collection were interviewed. Consequently, workers with more debilitating health conditions, including potentially severe cases of RSI/WMSDs, may have been systematically excluded due to their absence from work. Also, as with other cross-sectional studies, additional sources of bias must be considered, including those related to sampling process, interviewer performance and instrument administration. The methodological procedures adopted to minimize these potential biases are described in detail in Cremonese et al. (18).

Having outlined the study's limitations, its contributions to both academia and the workers' health care network deserve emphasis. To our knowledge, this is the first national and possibly international survey to describe the recent history of RSI/WMSD diagnoses and potential associated factors among beach workers. The stratification of results by sex provides novel insights and stimulates reflection on the differing ways the condition affects women and men. From methodological perspective, potential interviewer bias was minimized by employing a small, consistent team of 8–10 interviewers, conducting biweekly training and retraining sessions, preparing detailed reports and reviewing responses daily. Moreover, interviewer administration of the instruments, rather than self-administration further reinforced the quality and reliability of the data. With respect to the outcome, assessing RSI/WMSD diagnosis within the past 12 months favored a relatively short recall period. While this approach may have slightly underestimated prevalence, it helped reduce recall bias. Finally, we hope these findings will extend beyond academic discourse, fostering broader reflection on the working conditions on beach workers in Bahia and other similar environment worldwide. Most importantly, they should serve as catalyst for concrete actions to improve worker health and safety.

### Conclusions

This study provides novel evidence on the prevalence of RSI/WMSDs among beach workers in Salvador, Bahia, Brazil. Of the workers interviewed, 18.1% reported a diagnosis of RSI/WMSDs in the preceding year. Female workers were 1.67 times more likely to report the outcome than their male counterparts. Longer daily working hours were also associated with increased risk: participants working ≥13 h a day were 2.27 times more likely to report RSI/WMSDs compared with those working ≤ 8 h per day. Also, experiencing at least one WA in the past year increased the likelihood of RSI/WMSDs by 64%. Although no statistically significant association was detected, it is plausible that contextual factors such as high informality, heavy load carrying, lack of proper storage facilities for work equipment and lack of formal training contribute to the elevated prevalence observed. These findings highlight the need for targeted strategies by policymakers and occupational health authorities to improve working conditions and ensure comprehensive health protection for this still largely neglected workforce.

## Data Availability

The datasets presented in this study can be found in online repositories. The names of the repository/repositories and accession number(s) can be found below: https://doi.org/10.5281/zenodo.16636781.
